# The Carotenogenic* Dunaliella salina* CCAP 19/20 Produces Enhanced Levels of Carotenoid under Specific Nutrients Limitation

**DOI:** 10.1155/2018/7532897

**Published:** 2018-04-30

**Authors:** Sushanta Kumar Saha, Naresh Kazipet, Patrick Murray

**Affiliations:** Shannon Applied Biotechnology Centre, Limerick Institute of Technology, Moylish Park, Limerick V94 E8YF, Ireland

## Abstract

*Dunaliella salina* is the popular microalga for *β*-carotene production. There is still a growing demand for the best strain identification and growth conditions optimization for maximum carotenoids production. Some strains are noncarotenogenic while other strains may respond differently to applied growth conditions and produce enhanced carotenoid levels. This study tested the carotenogenic ability of* Dunaliella salina* CCAP 19/20 under sixteen stress conditions and certain biochemical changes in response to specific stress were investigated. This study identified the above strain as carotenogenic, which produces maximum carotenoids under high light (240 *μ*mol photons m^−2^ sec^−1^) when combined nitrogen and micronutrients (Cu or CuMn) were limited. Based on the intensity of extracted ions chromatograms, lutein (*m/z* 568.4357) appears as the major carotenoid followed by *β*-carotene (*m/z* 536.4446) and *α*-carotene (*m/z* 536.4435). A polypeptide of 28.3 kDa appeared while another polypeptide of 25.5 kDa disappeared in stress cells as compared to noncarotenogenic cells. Expression levels of antioxidative-enzyme superoxide dismutase-1 (SOD1, H_2_O_2_-resistant) remained identical, while the prominent H_2_O_2_-sensitive isoforms SOD2 and SOD3 were downregulated during carotenogenic conditions. Overall, increased carotenoids levels might be due to the response of differential expression of specific polypeptides and retention of H_2_O_2_-resistant SOD, which eventually might help the organism to thrive in the tested stress conditions.

## 1. Introduction

*Dunaliella salina* is a marine, unicellular, oxygen-evolving eukaryotic microalga (Chlorophyceae, Dunaliellales), which scavenges atmospheric CO_2_ through photosynthesis and grows as motile biflagellated green cells under favourable growth conditions. Morphologically identical* Dunaliella* strains may be biochemically different with noncarotenogenic and carotenogenic nature [1, 2]. Under stressful environmental conditions, such as nitrogen limitation, low temperature, high irradiance, or high-salt concentrations,* D. salina* cells accumulate carotenoids [3–6]. Nitrogen limitation and high irradiance were reported as favourable *β*-carotene accumulation factors in* D. salina* [7]. Carotenoids are accessory light-harvesting pigments that are structural components for photosystem assembly and help in protecting the photosynthetic apparatus from oxidative damage caused by ROS [8, 9]. The production of ROS is induced due to various stress growth conditions [10, 11]. However, microalgae have enzymatic and nonenzymatic prosurvival mechanisms that either scavenge the produced ROS or inhibit the production of ROS [3]. Excessive ROS might otherwise damage irreversibly lipids, proteins, and DNA molecules ultimately leading to cell death [12]. ROS and autophagy are associated with cell death, which eventually helps in recycling macromolecules (amino acids, lipids, and sugars) for temporal adaptation to adverse conditions [13]. The carotenoid *β*-carotene (nonenzymatic mechanism) protects the cells by quenching the harmful effect of ROS and free radicals generated due to oxidative stress [14].* D. salina* is the major source of natural antioxidants (*β*-carotene and lutein) that have applications in food, feed, and cosmetic and health industries [9, 15]. Noncarotenogenic* Dunaliella* strains nowadays receive attention for biofuel production because of their fast growth and lipid production ability [2].

Like higher plants, microalgae also have evolved multiple strategies to survive oxidative stress caused due to suboptimal growth conditions. Under high-light irradiance, the reaction center proteins and the core chlorophyll-protein-antenna complex change suitably to protect microalgae cells from photoinhibition [16]. In higher plants, the excess photons energy is dissipated through nonphotochemical quenching by specific carotenoid production and thus ROS generation is minimized [17]. Cellular antioxidant carotenoids content increases and the activities of the antioxidative-enzyme superoxide dismutase (SOD) also change significantly during stress conditions [4, 10, 18, 19]. SODs are of four types depending on their metal prosthetic groups, such as Mn-SOD, Fe-SOD, and Cu/Zn-SOD [10, 20]. SOD (enzymatic mechanism) dismutates superoxide radicals to hydrogen peroxide (H_2_O_2_) and the concentration of H_2_O_2_ may regulate the activities of SOD isoenzymes. For example, Mn-SOD is resistant to certain concentration of H_2_O_2_* in vitro*, while Fe-SOD and Cu/Zn-SOD are sensitive at the same concentration of H_2_O_2_ [20]. The activities of these four types of SODs may therefore be dependent on intracellular H_2_O_2_ concentration as well as availability of metal prosthetic groups that is supplied as micronutrients in the growth medium [21]. Therefore, a strong oxidative stress condition may be created which would enhance carotenoid biosynthesis as an alternative prosurvival strategy to survive the stress conditions applied.

*Dunaliella salina* is the popular microalga known to produce *β*-carotene; however there is a growing need for identification and growth optimization for the best strain suitable for variable cultivation conditions. Some strains may be efficient in responding to applied growth conditions and enhance carotenoid production while other strains may not be carotenogenic at all. This study was undertaken to identify if* D. salina* CCAP 19/20 is carotenogenic and if so, how its carotenoid levels are enhanced under selected stress conditions. Other biochemical changes were also investigated to understand the response of major antioxidative-enzyme superoxide dismutase and stress-related proteins expression. These data would help in growth optimization of* D. salina* CCAP 19/20 for maximum carotenoid production.

## 2. Materials and Methods

### 2.1. Green-Phase and Stress-Phase Culturing of Microalga

*Dunaliella salina* CCAP 19/20 (hereafter* D. salina*) was obtained from the Culture Collection of Algae and Protozoa, SAMS Research Services Ltd., UK. The microalga was maintained in modified ATCC 1174DA medium (1 M NaCl, 20 mM NaHCO_3_, 5 mM KNO_3_, 5 mM MgSO_4_, 0.6 mM CaCl_2_, 0.2 mM KH_2_PO_4_, 2 *μ*M FeCl_3_, 6 *μ*M EDTA, 100 *μ*M H_3_BO_3_, 7 *μ*M MnCl_2_, 0.8 *μ*M ZnCl_2_, 0.03 *μ*M CoCl_2_, and 0.4 *μ*M CuSO_4_) at low-light intensity of 40 *μ*mol photons m^−2^ sec^−1^ at 20°C. For actively growing green-phase cells and growth performance, the microalga was cultured in 250 ml Erlenmeyer flasks containing 100 ml of modified ATCC 1174DA medium for 10 and 17 days, respectively. Briefly, 500 *μ*l of healthy green cells was inoculated per 100 ml growth medium to obtain an initial (day 0)* in vivo* absorbance of cells of ~0.03 at 665 nm and 680 nm. The flasks were incubated in an environmental growth chamber at 22°C, under the PAR (photosynthetically active radiation, 400–700 nm) illumination 85 *μ*mol photons m^−2^ sec^−1^ (low-light, LL) for 16/8 h light/dark cycle with shaking at 150 rpm. The active green-phase growth of microalga was also tested under medium light (ML, 120 *μ*mol photons m^−2^ sec^−1^) and high-light (HL, 240 *μ*mol photons m^−2^ sec^−1^) conditions. On the 10th day of growth, green cells were harvested and either retained as control cells for proteins and enzyme analysis or used as inoculum for stress-phase cultivation up to 14 days. Initial pH of the green-phase or stress-phase cultivation medium was set at 7.7 ± 0.02, which increased to 8.2–8.3 on 14th day of cultivation.

A total of sixteen stress conditions were tested to identify the best stress condition for maximum carotenoid production. Ten-day-old green-phase cells were harvested carefully and quickly by centrifugation at 3000 ×g at 20°C for 4 min and were used to set up the stress experiments. The pellets obtained after centrifugation were washed twice with stress medium devoid of all micronutrients and KNO_3_. Finally, the pellets were resuspended in 3 ml of the above stress medium. Stress experiments were carried out in triplicate in 24-well cell culture plates. Each well containing 2 ml of appropriate stress medium was inoculated with 50 *μ*l of the above resuspended cells. The modified ATCC 1174DA medium without KNO_3_ was used as control stress medium. The remaining fifteen stress media were based on control stress media plus elimination of one or more micronutrients. For the stress growth, plates were incubated at 22°C, under the high-light PAR illumination of 240 *μ*mol photons m^−2^ sec^−1^ with 16/8 h light/dark cycle. Each day cells were mixed gently by pipetting which helped in synchronous stress development and prevented condensation of water on the lids.

### 2.2. Biomass for Protein and Enzyme Analysis

After analysis of carotenoids content for 14 days of stressed cells, only three stress conditions (control stress, Cu^−^, and Cu^−^Mn^−^) and control green-phase condition were chosen for protein and SOD isoenzymes profile analysis. Cells required for protein extractions were obtained from harvesting 7-day-old cultures grown in 250 ml Erlenmeyer flasks (in triplicate) containing 100 ml of appropriate medium. These stress cultures were incubated at 22°C, under the PAR illumination of 240 *μ*mol photons m^−2^ sec^−1^ with 16/8 h light/dark cycle and shaking at 150 rpm.

### 2.3. Growth and Pigment Content

Active green-phase growth of* D. salina* was monitored by change in* in vivo* absorbance of cells at 665 nm and 680 nm. For* in vivo* absorbance as an indicator of cell density, 100 *μ*l of uniformly suspended cells from specific time-points of growth was transferred to a 96-well plate reader (BioTek Synergy 4). The absorbance value was recorded quickly prior to settling of cells. Cell dry weight of pelleted biomass was estimated gravimetrically after overnight oven dry at 55°C. Cellular growth during stress-phase was monitored by estimating the pigments content from indicated time points. For spectrophotometric measurement of chlorophylls and total carotenoids, 200 *μ*l of cells was pelleted by centrifugation at 10,000 ×g for 2 min and the pellets were resuspended uniformly in 200 *μ*l of dimethyl sulfoxide (DMSO) prior to extraction in the dark at 55°C in a water bath. The extracts were centrifuged at 15,000 ×g for 3 min and the absorbance of the clear supernatant was recorded at 480, 649, and 665 nm, in a 96-well plate reader (BioTek Synergy 4) to estimate the content of total carotenoid and total chlorophyll following the equations of Wellburn [22].

### 2.4. Carotenoids Analysis by LC/MS

Analysis of carotenoids by LC/MS was carried out as per protocol described earlier [23]. Briefly, supernatants of DMSO extracts used for spectrophotometric determination were stored at −20°C and were thawed only on the day of LC/MS run. Freeze-thawed supernatants were centrifuged at 13,000 ×g for 6 min and transferred to amber vials. 10 *μ*l of clear supernatants was injected onto an Agilent 1260 series HPLC connected to Q-TOF mass spectrometer (Agilent 6520). Carotenoids were resolved by Agilent Zorbax Eclipse C-18 (2.1 mm × 150 mm × 3.5 *μ*m) column and gradient elution solvents. Methanol, methyl tert-butyl ether (MTBE), and H_2_O (90 : 5 : 5) served as mobile phase A, while mobile phase B contained a solution of methanol, MTBE, and H_2_O (43 : 55 : 2). Formic acid (0.1%, v/v) was used as an additive in both the mobile phases. The column eluents were directed through a diode array detector (DAD), which was monitored at 478 nm. Finally, 100% of the eluent was passed through the electrospray ionisation source of the Q-TOF mass spectrometer to record the* m*/*z* values from 50 to 1700 and the scanning was performed in positive ionisation mode. *β*-Carotene (Sigma) was used as standard to validate the LC/MS method for carotenoids analysis by comparing the retention time (RT) and specific mass for *β*-carotene (*m/z* 536.4446). Other carotenoids present in the samples were identified based on the accurate mass of the known compound.

### 2.5. Protein and Enzyme Electrophoresis

Samples for protein profile and assay of enzyme activities were prepared from thoroughly washed* D. salina* cells rewashed with extraction buffer (62.5 mM Tris-Cl, pH 6.8). Cell pellets were homogenized in a prechilled mortar and pestle in presence of glass powder (~0.5 mm size, clean and sterile) and ice-cold extraction buffer. The extracts were centrifuged at 15,000 ×g for 15 min and the process was repeated twice to obtain clear supernatants. The amounts of total soluble proteins were estimated by Bradford reagent, B6916 (Sigma), using BSA as standard [24] and used for both sodium dodecyl sulfate-polyacrylamide gel electrophoresis (SDS-PAGE) and native-PAGE for activity staining of superoxide dismutase.

Electrophoresis was carried out at room temperature (~20°C) and in ice bath (~4°C) for SDS-PAGE and native-PAGE, respectively, with a 1 mm thick polyacrylamide gel in Tris-glycine buffer (pH 8.3). In the case of SDS-PAGE, SDS was included in the running buffer (0.1%) as well as in sample buffer (10%). For SDS-PAGE, 2 *μ*g of total proteins was boiled for 3 min with sample buffer and centrifuged briefly before loading. For native-PAGE, 8 *μ*g of total proteins was loaded in each well along with native sample buffer without any denaturing agents. Samples were then electrophoresed at 100 V for 30 min through the stacking gel (6%) and by separating gel (10%) at 120 V for 1 hour and at 140 V until the tracking dye reached the bottom of the gel.

### 2.6. SDS-PAGE Gel Staining

After electrophoresis, the gel was washed twice, each for 10 minutes with Milli-Q water, and then stained overnight with GelCode blue stain reagent (Thermo Scientific, USA) and destained with distilled water before documentation.

### 2.7. Native-PAGE Gel Staining

Activity staining for superoxide dismutase (SOD) on gel [25] and their sensitivity to H_2_O_2_ assay were carried out as described earlier [20]. Briefly, one part of the gel was incubated in 50 mM Tris-Cl (pH 8) containing 5 mM H_2_O_2_ (test) and another part was incubated in 50 mM Tris-Cl (pH 8) containing no inhibitors (control) for 20 min in the dark. Then, both gels were transferred to staining solution (50 ml of 50 mM Tris-Cl (pH 8), 10 mg NBT, 1 mg EDTA, and 2 mg riboflavin) and soaked for 30 min in the dark at room temperature (~20°C). Finally, gels were illuminated with white fluorescent light (120 *μ*mol photons m^−2^ s^−1^) for a maximum of 30 min to develop the achromatic bands due to SOD activities.

### 2.8. Gel Documentation and Statistical Analysis

Gel images were captured with 10-megapixel automatic digital camera with ISO Max set up at 400 (Lumix DMC-FS7, Panasonic) on a white fluorescent light box and alternately gel image was captured by CCD camera for the analysis of protein by Total Lab TL100 software (DNR Bio-Imaging System, Israel). Gel images were processed by improving the brightness contrast of specific regions by using the image analysis software InkScape for obtaining relative staining intensity. 1D gel analysis was performed using “First Order Lagrange” type of curve and propagating *R*_*f*_ of the standard molecular weight protein marker (Broad Range, New England Biolabs) to determine the molecular weight of unknown protein bands. Data presented in the graphs were averages of three replicates ± standard deviation.

## 3. Results

### 3.1. Active Green-Phase Growth

**Figure 1:**
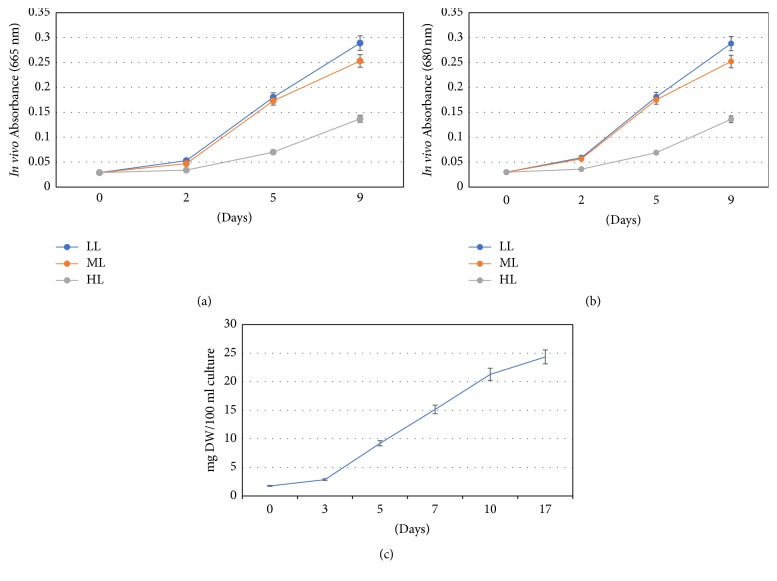
Green-phase growth under low light (LL), medium light (ML), and high light (HL) as monitored by change in* in vivo* absorbance at 665 nm (a) and at 680 nm (b). Change in cell densities under optimum low-light condition as estimated by cell dry weight (c).

*D. salina* was grown as active green-phase cells under moderate light illumination (85 *μ*mol photons m^−2^ sec^−1^) in modified ATCC 1174DA medium containing all essential micro- and macronutrients. However, cell growth under three light regimes (LL, ML, and HL) was monitored by* in vivo* optical density of cells at 665 nm and 680 nm (Figure 1). The highest absorbance values were recorded for LL followed by ML and HL. There was an increase of absorbance of 9.96- and 9.6-fold, respectively, at 665 nm and 680 nm under low-light condition on day nine compared to the cells inoculated on day zero (Figure 1). Growth performance of* Dunaliella salina* CCAP 19/20 under low light was tested in terms of change in cell dry weight per 100 ml of cultures. Under optimum laboratory growth conditions, a maximum of approximately 25 mg dry weight biomass was obtained on the 17th day of growth with about 14-fold increase compared to the cells inoculated on day zero (Figure 1).

### 3.2. Stress Growth and Change in Chlorophyll Content

**Figure 2:**
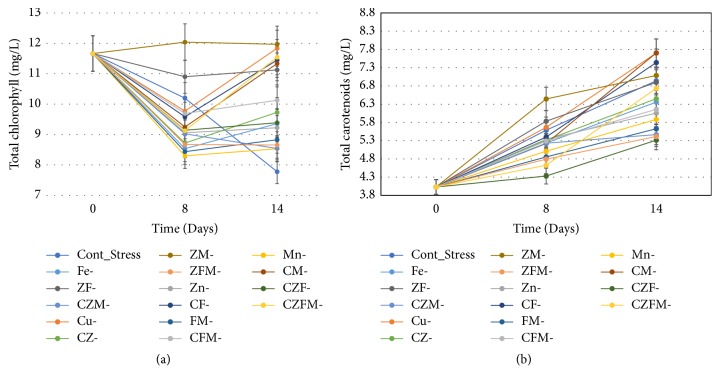
Change in total chlorophyll (a) and total carotenoids (b) content of* Dunaliella salina* CCAP 19/20 during stress-phase culturing under high-light intensity in micronutrients depleted media. Cu (C), copper; Zn (Z), zinc; Mn (M), manganese; Fe (F), iron.

Figure 2(a) shows the change in total chlorophyll content of* D. salina* during stress phase of cultivation. For carotenogenesis, actively grown green-phase cells were transferred to medium devoid of combined nitrogen and devoid of specific micronutrients before growing under high-light (240 *μ*mol photons m^−2^ sec^−1^) incubation for stress-phase cultivation. The complete medium devoid of combined nitrogen served as control stress medium. There was sharp decline in total chlorophyll content on day 4 in all conditions, while in few conditions (such as ZM^−^, Cu^−^, CZFM^−^, CF^−^, ZF^−^, and CM^−^) there was gradual increase in chlorophyll content essentially up to the amount present on day 0 of stress-phase cultivation. The chlorophyll content of almost half of the stress conditions tested declined only up to 50% on day 14 compared to day 0 of stress cultivation.

### 3.3. Stress Growth and Change in Carotenoid Content

Cellular total carotenoids content began increasing when the actively grown green-phase cells were transferred to any of the tested stress media and were incubated under high-light intensity (240 *μ*mol photons m^−2^ sec^−1^). However, only one-third of the tested stress conditions showed substantial increase in total carotenoids content (Figure 2). The cell mass colour was golden-yellowish compared to actively grown green cells; however, the microscopic observation of cells revealed only yellowish-green cytoplasmic content (Supplementary Fig.  1). Of the sixteen stress conditions tested, the highest carotenoid content on day 14 was recorded only in two stress conditions: (i) stress medium devoid of KNO_3_ and copper and (ii) stress medium devoid of KNO_3_, copper, and manganese (Figure 2). The control stress condition and another stress medium devoid of KNO_3_, copper, and iron were the next best stress conditions in terms of carotenoid content. The maximum increase in carotenoid content was 91% in two best conditions, while the increase in carotenoid content in control stress condition was 72.63% on day 14 compared to day zero of stress.

**Figure 3:**
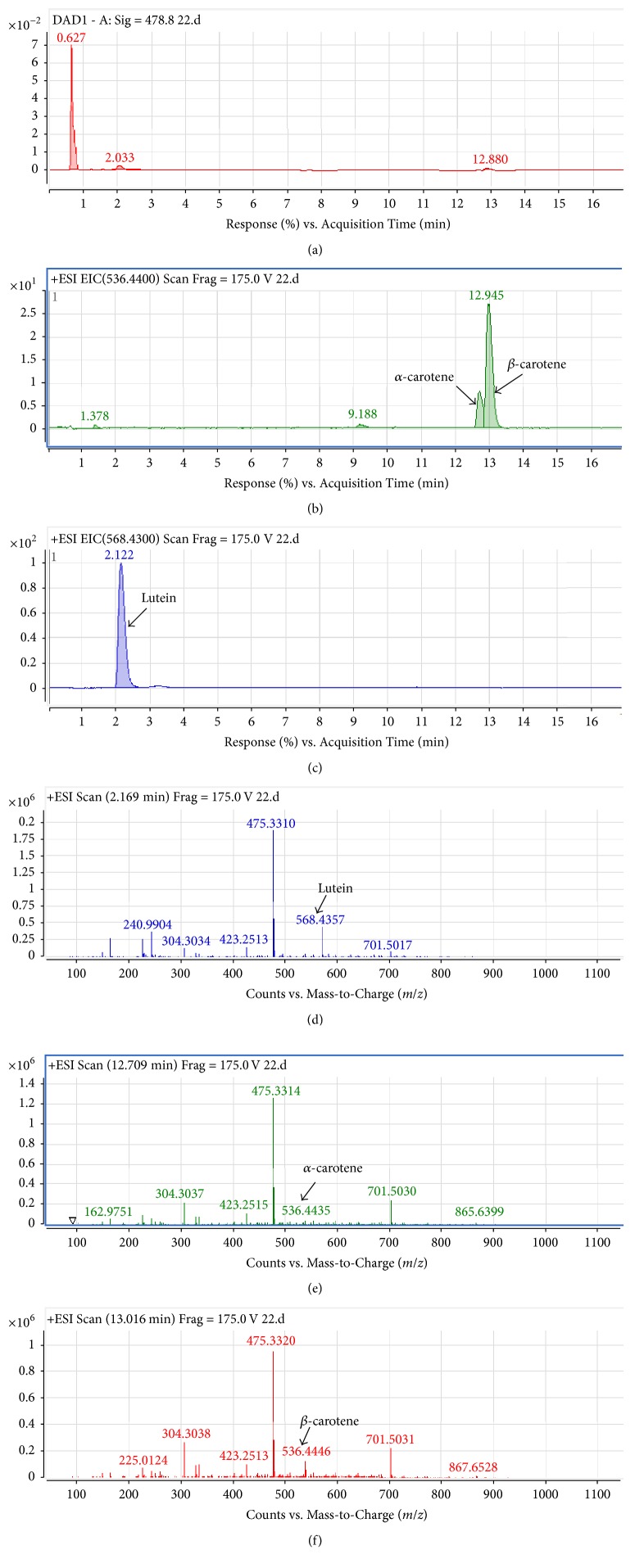
LC-MS analysis of carotenoids from* Dunaliella salina* grown for nine days under high light in control stress medium. The diode array detector (DAD) profiles of carotenoid pigments monitored at 478 nm (a); extracted ion chromatograms (EIC) showing the peaks of *α*- and *β*-carotene (b) and lutein (c); mass spectrometric identification of lutein (*m/z* 568.4357, (d)), *α*-carotene (*m/z* 536.4435, (e)), and *β*-carotene (*m/z* 536.4446, (f)).

The carotenoid profile (of DMSO extract of control stress grown cells) obtained by diode array detector (DAD) monitoring at 478 nm showed three detectable peaks at 0.627, 2.03, and 12.88 min (Figure 3(a)). Extracted ion chromatograms (EICs) with specific mass for known carotenoids and their mass spectra profile identified lutein (2.12 min,* m/z* 568.4357), *β*-carotene (12.94 min,* m/z* 536.4446), and *α*-carotene (12.7 min,* m/z* 536.4435) (Figures 3(b)–3(f)). Considering the peak areas of EIC chromatograms, it appears that lutein (peak area 6445806.87) is the most abundant carotenoids followed by *β*-carotene (peak area 1574785.62) and *α*-carotene (peak area 418304.25) (Figures 3(b) and 3(c)).

### 3.4. Chlorophyll : Carotenoid Ratio

**Figure 4:**
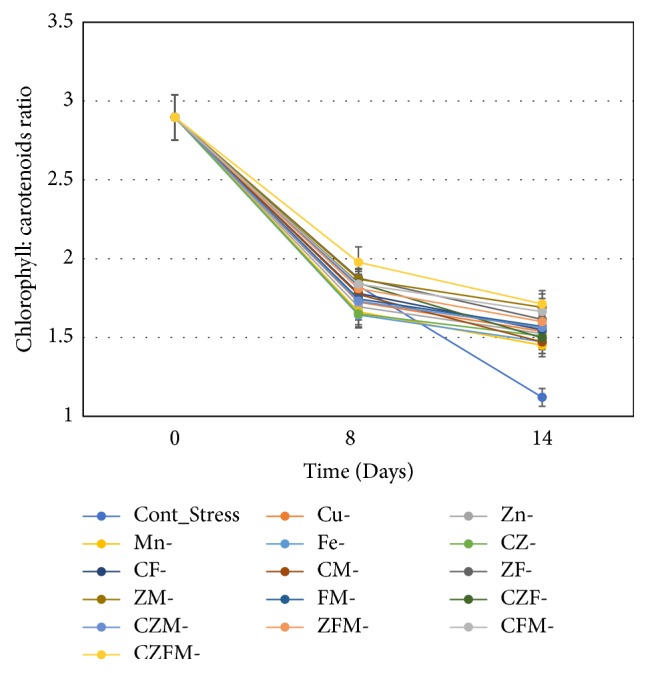
Gradual decrease in chlorophyll : carotenoids ratio during stress-phase culturing of* Dunaliella salina* CCAP 19/20 suggesting their pigments status. Cu (C), copper; Zn (Z), zinc; Mn (M), manganese; Fe (F), iron.

In several microalgae, the process of carotenogenesis is two-phase growth dependent and the ratio of chlorophyll to carotenoid concentrations particularly during their stress-phase growth has been used as indicator of optimum stress conditions [26]. In this study, this ratio was reduced from 2.9 up to 1.12 in stress conditions on day 14 indicating a good stress response with the increase of carotenoid production in almost all tested stress conditions and corresponding decrease in chlorophyll content (Figures 2 and 4). However, only control stress condition was best in terms of lowest chlorophyll-to-carotenoid ratio (Figure 4).

### 3.5. Superoxide Dismutase Isoenzyme Profile

**Figure 5:**
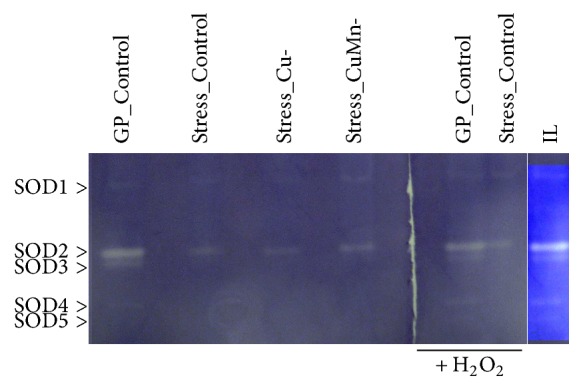
Activity staining of superoxide dismutase isoenzymes and their sensitivity to H_2_O_2_. The source of enzymes was* Dunaliella salina* CCAP 19/20 cells from active growth as well as stress growth phases. GP_Control, green-phase control cells; Cu, copper; Mn, manganese; IL, improved lane (H_2_O_2_ treated GP_control lane) showing five achromatic bands after adjustment of brightness and contrast of selected region of the gel.

Superoxide dismutase (SOD) is the first line of enzyme defence system. The activity staining profile of SOD showed significant variations between the enzymes of green-phase and stress-phase grown cells of* D. salina* (Figure 5). A total of five isoenzyme bands (SOD1-SOD5) were detected. The expression of SOD1 was essentially not affected in any of the stress conditions as the achromatic zones in all lanes were similar to that of green growth phase cells. The prominent isoforms SOD2 and SOD3 were downregulated in all stress conditions. The levels of expression of SOD2 in stress conditions were one-third and SOD3 were negligible compared to green growth phase cells. The expression of less prominent isoforms SOD4 and SOD5 that were present in green growth phase cells completely disappeared in all stress conditions.

Assay for H_2_O_2_ sensitivity was carried out by treating part of the gel (right gel, Figure 5) with 5 mM H_2_O_2_ prior to standard SOD activity staining. The parallel comparison of treated and untreated gels revealed that the prominent isoforms SOD2 and SOD3 from green-phase cells (GP_control) were slightly sensitive to H_2_O_2_. SOD2 from stress condition (Control_stress) was slightly less sensitive to H_2_O_2_ while SOD3 from stress condition (Control_stress) was highly sensitive to H_2_O_2_ treatment.

### 3.6. Protein Profile

**Figure 6:**
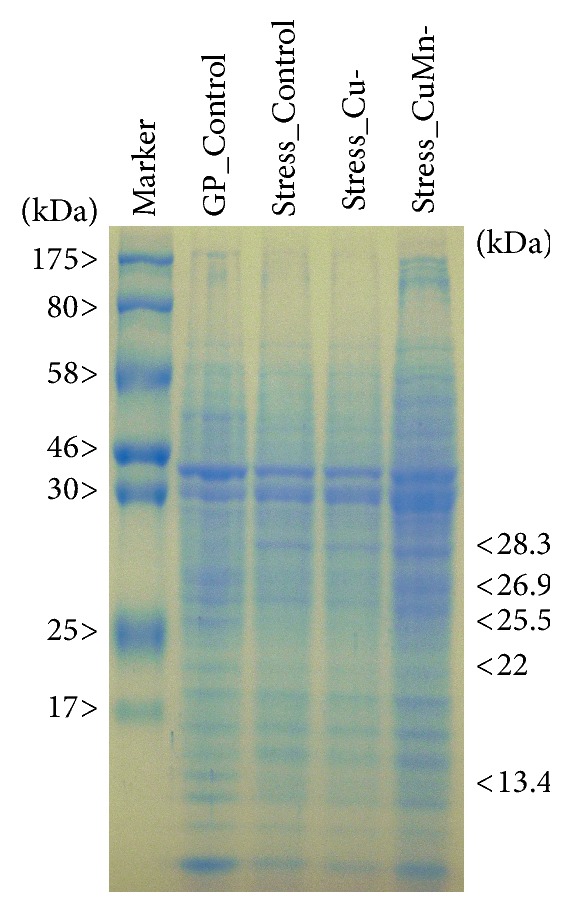
Modification of protein synthesis in* Dunaliella salina* CCAP 19/20 during stress growth conditions of macro- and micronutrients depletion. Protein samples (2 *μ*g), from healthy green cells actively grown for 5 days in complete medium (modified ATCC 1174DA) (GP_control), in stress medium (complete medium devoid of KNO_3_) (Control_stress), and in two other stress media (complete medium devoid of Cu or CuMn in addition to KNO_3_ depletion), were resolved by SDS-PAGE (10%) and visualized by colloidal Coomassie dye G-250 staining. To determine the molecular weights of prominent polypeptides either missing, appearing new, or at enhanced levels, a broad-range proteins molecular weight mixture (New England Biolabs) were run simultaneously.

The protein profiles of* D. salina* cells from all three stress conditions [KNO_3_^−^ (Control stress), KNO_3_^−^ and Cu^−^ (Stress-Cu), and KNO_3_^−^ and CuMn^−^ (Stress-CuMn)] differed markedly from those of actively grown green-phase cells (Figure 6, GP_control). A polypeptide of 28.3 kDa appeared in cells under all stress conditions, which was absent in green-phase growth cells, while a polypeptide of 25.5 kDa present in green-phase growth cells disappeared under all stress conditions (Figure 6). Three polypeptides of 26.9, 22, and 13.4 kDa, found in green growth phase cells, were differentially expressed in all stress conditions as visualized by variable staining intensities (Figure 6).

## 4. Discussion

Some* Dunaliella* sp. produce commercially important carotenoid pigments and their levels were found to be enhanced under specific stress conditions, while there are some* Dunaliella* sp. that does not show induced carotenogenesis. In this study, a strain of* Dunaliella salina* CCAP 19/20 was characterized for its carotenogenic ability under sixteen stress conditions. Initially, active green-phase growth was tested under low-, medium-, and high-light conditions, and the tested strain performed better under low-light (85 *μ*mol photons m^−2^ sec^−1^) condition with about 14-fold biomass increase on the 17th day of growth (Figure 1). The percentage increase of biomass per day of this* Dunaliella salina* strain (CCAP 19/20) appears much higher compared to* Dunaliella salina* CCAP 19/18 [18]. The active green cells responded immediately to stress growth conditions with sharp decline of chlorophyll content, possibly due to both photooxidation of photosystem II reaction centers and electrochemical gradients formation on the transmembrane after the onset of high-light illumination as reported in microalga* Haematococcus pluvialis* [27]. Generally, the chlorophyll content reduces as a common stress response of high-light and nitrogen limitation as reported in green microalgae* Haematococcus pluvialis* [28, 29] and* Dunaliella bardawil* [5]. However, the unusual increase in chlorophyll content in certain stress conditions in this strain can be hypothesized to be due to (1) acclimation to the stress environment by increased oxidative stress tolerance [30], (2) recovery of high-light stressed cells through dark cycle repair as reported in* Scenedesmus vacuolatus*, where heat treated cells with complete inhibition of photosynthesis were recovered during dark recultivation [31], and (3) mere availability of macromolecules through recycling of dead organic matters released to the culture medium due to excess ROS and autophagy [13]. Another study found the transient increase in cell division in* D. salina* under nitrogen depletion at a constant light regime in a turbidostat and eventually increased *β*-carotene as well as biomass yield [32].

The strain of* Dunaliella salina* CCAP 19/20 could be identified as carotenogenic. The cellular carotenoids content increased in all stress growth conditions at high-light intensity (240 *μ*mol photons m^−2^ sec^−1^), while the substantial increase in total carotenoids was found only in one-third of the stress conditions tested (Figure 2). The data suggest a positive effect on carotenoid content due to removal of copper and KNO_3_ from the stress growth conditions. There are several reports on increased carotenoids content in microalgae in response to high-light and combined nitrogen depletion (*D. bardawil* [5] and* H. pluvialis* [29]). There was another report on* D. salina* strain that died under high-light illumination due to reduced chlorophyll content with slow growth rate [5]. In contrast, the carotenoid content of a cyanobacterium* Oscillatoria willei* BDU 130511 was reduced even under low-light incubation in nitrogen-limited growth medium [10]. Detail analysis of carotenoids profile using LC-MS revealed that the major carotenoids are lutein, *β*-carotene, and *α*-carotene in the* D. salina* CCAP 19/20 strain. The data of this study is in agreement with previous reports, where lutein was identified as one of the major carotenoids in* D. salina* [33] and in* D. tertiolecta* [34]. However,* D. salina* is mainly known for its highest *β*-carotene content [6, 35] and a relatively low amount of *α*-carotene in certain* D. salina* strains under specific growth temperatures [35]. A recent study however demonstrated the modified carotenoid production pathways in* Dunaliella salina* by using cyclization inhibitors 2-methylimidazole (2MI) and 3-amino-1,2,4-triazole (Amitrol). The study found that 2MI was effective in lutein accumulation without affecting cell viability of* D. salina* when grown at high temperature [36].

Most carotenogenic microalgae decline their chlorophyll content while gradually increasing the content of carotenoids upon transfer to stress growth conditions and the ratio of chlorophyll to carotenoid has been used as optimum stress indicator. The rate of reduction of chlorophyll-to-carotenoids ratio in* Dunaliella* strain (CCAP 19/20) is low. It was reported in another study that five out of the seven strains of* D. salina* showed a relatively reduced chlorophyll-to-carotenoid ratio in low-light (40 *μ*mol photons m^−2^ sec^−1^) compared to high-light (110 *μ*mol photons m^−2^ sec^−1^) condition at the highest growth temperatures of 26°C [35]. In* Dunaliella bardawil*, chlorophyll-to-*β*-carotene ratio was found inversely proportional to the growth irradiance during cell division cycle [5]. The chlorophyll-*a*-to-total carotenoid ratio was indicative of good physiological response in green microalga* H. pluvialis* during nitrogen depletion stress [26].

The SOD isoenzyme profile possibly explains differential response to cellular H_2_O_2_ environment especially under stress conditions. The less prominent isoform SOD1 from both green-phase and stress-phase cells was resistant to H_2_O_2_ treatment and hence this could be Mn-SOD. Retention of Mn-SOD in all growth conditions might suggest a specific requirement to protect against respiratory ROS generated in the mitochondrial membranes [10, 37]. H_2_O_2_ sensitive bands of* D. salina* could be Fe-SOD whose levels were found reduced in all the stress conditions. This possibly suggests toxic levels of H_2_O_2_ production internally, which after release to the culture medium may aid in degrading organic matter for nutrients recycling [10]. Increase in H_2_O_2_ content is a common consequence of abiotic stresses including salinity stress in* D. salina* [4]. Previous studies suggest that Fe-SOD may protect cyanobacteria against cytosolic superoxide ions generated during oxidative stress due to nitrogen limitation [10, 38]. Therefore, this microalga might have adopted an alternative stress alleviating mechanism to protect against harmful effects of cytosolic superoxide ions. Heat shock protein 90 (Hsp90) of* Dunaliella salina (dshsp90)* was found to be upregulated due to heat and salt shock suggesting that* dshsp90* might serve as early marker of environmental stress response in* D. salina* [39]. The SDS-PAGE protein profiles of green phase and stress phase are essentially different. Particularly, the three polypeptides (25.5, 26.9, and 28.3 kDa) could actually be LHC-II (light-harvesting complex II) polypeptides, which may contain a minimum of six protein subunits ranging within 25–31 kDa [40]. Two polypeptides of 22 and 13.4 kDa could be copper-responsive proteins involved in electron transfer and redox reactions. Copper-responsive proteins of 5–25 kDa were reported in germinating embryos of* Oryza sativa* [41]. Earlier, a 22 kDa ferredoxin-binding protein was suspected to be modified to 28 kDa in* D. salina* under high-light irradiance as detected immunologically [16]. A recent study on* Dunaliella parva* found a total of 227 upregulated proteins and 159 downregulated proteins under nitrogen-limited stress condition. Functional analyses of the above differentially expressed proteins suggested that nitrogen limitation could alter the levels of proteins expression related to photosynthesis, stress response, lipid metabolism, carbohydrate metabolism, and nitrogen metabolism in* Dunaliella parva* [42].

Overall, the differential protein profile suggests photooxidative stress response of* D. salina* CCAP 19/20. The sharp decline of chlorophyll levels and then gradual increase of carotenoids particularly lutein and the differential response of SOD might explain the survival strategies of* D. salina* CCAP 19/20 under the tested stress conditions. As a short-term strategy to survive under oxidative stress conditions, microalga* H. pluvialis* showed differential expressions of SOD isoenzymes, while as long-term survival strategy the organism may adopt chronic molecular defence strategies by carotenoid accumulation [37].

## 5. Conclusions

The carotenogenic* D. salina* CCAP 19/20 produces enhanced level of carotenoids including lutein and *β*-carotene when cultivated in growth medium devoid of specific micronutrients (Cu or CuMn) and nitrogen under high-light (240 *μ*mol photons m^−2^ sec^−1^) illumination. The response of chlorophyll and carotenoid pigments and differential expression of SOD isoenzymes as well as proteins related to photosynthesis helped the organism to survive tested stress conditions. The data obtained on various biochemical changes may be used as base for further evaluation of* D. salina* CCAP 19/20 for commercial suitability and growth optimization.

## Abbreviations

DMSO: Dimethyl sulfoxide
H_2_O_2_: Hydrogen peroxide
PAR: Photosynthetically active radiation
ROS: Reactive oxygen species
SOD: Superoxide dismutase.

## Supplementary Materials

Supplementary Materials Supplementary Fig. 1:* Dunaliella salina* CCAP 19/20 were grown as healthy green cells for generating biomass (first flask from left), which were then transferred to three stress media to obtain dark-yellowish carotenogenic cells (the remaining three flasks in the upper panel). Lower panel shows the photomicrographs (not in scale) of corresponding green-phase and stress-phase cells colour.
