# Supplementary figures and images for: The Carotenogenic Dunaliella salina CCAP 19/20 Produces Enhanced Levels of Carotenoid under Specific Nutrients Limitation
Source: Biomed Res Int. 2018 Apr 30;2018:7532897. doi: 10.1155/2018/7532897 (PMC5952566; doi:10.1155/2018/7532897)

## Slide 1
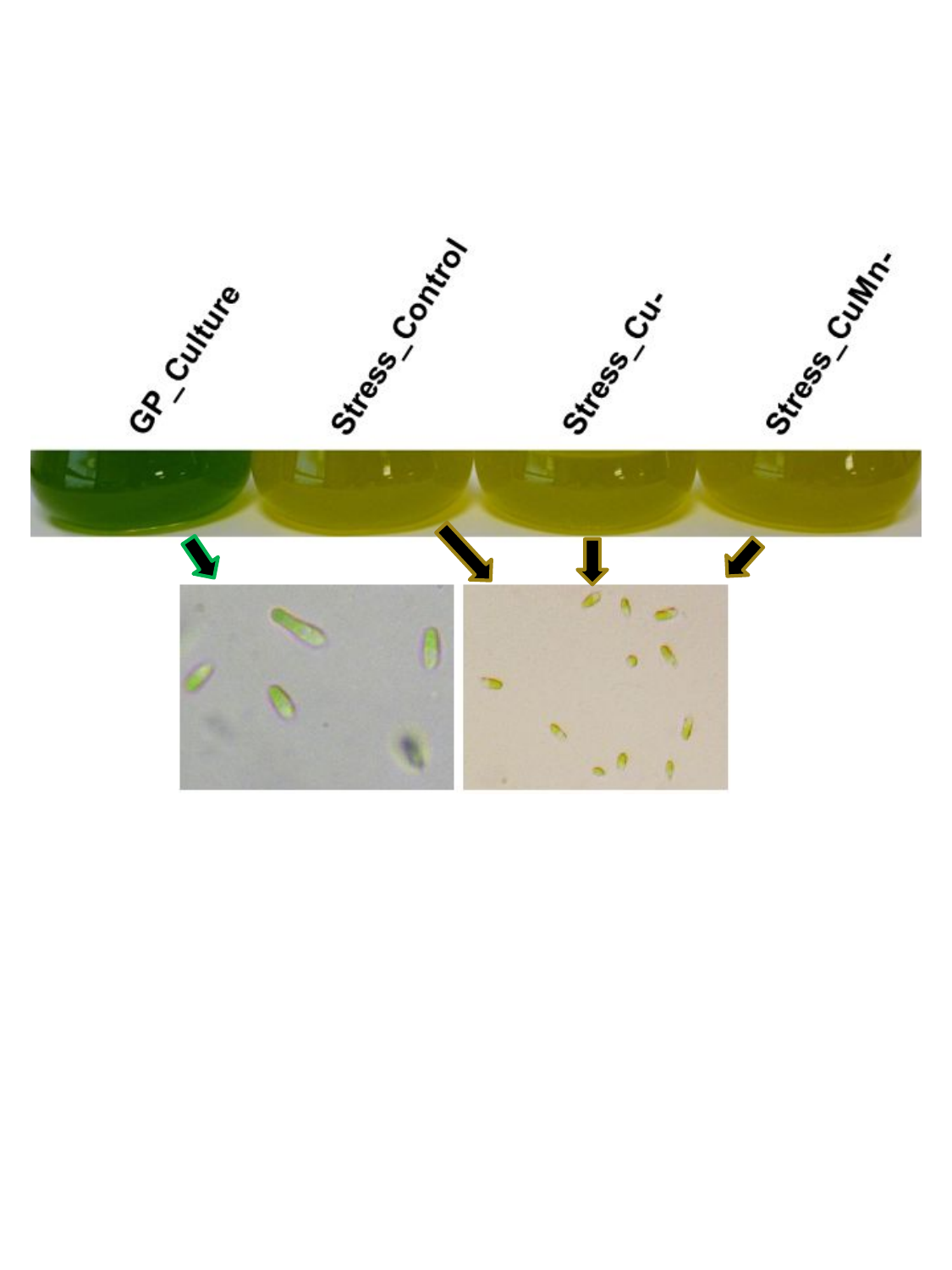

Supplement: Supplementary Materials — Supplementary Fig. 1: Dunaliella salina CCAP 19/20 were grown as healthy green cells for generating biomass (first flask from left), which were then transferred to three stress media to obtain dark-yellowish carotenogenic cells (the remaining three flasks in the upper panel). Lower panel shows the photomicrographs (not in scale) of corresponding green-phase and stress-phase cells colour. [file 7532897.f1.pptx]
